# Detection of Volatiles by HS-SPME-GC/MS and Biological Effect Evaluation of Buddha’s Hand Fruit

**DOI:** 10.3390/molecules27051666

**Published:** 2022-03-03

**Authors:** Sara Vitalini, Marcello Iriti, Elisa Ovidi, Valentina Laghezza Masci, Antonio Tiezzi, Stefania Garzoli

**Affiliations:** 1 Department of Agricultural and Environmental Sciences, Università degli Studi di Milano, 20133 Milan, Italy; sara.vitalini@unimi.it; 2 Phytochem Lab., Department of Agricultural and Environmental Sciences, Università degli Studi di Milano, 20133 Milan, Italy; 3 National Interuniversity Consortium of Materials Science and Technology (INSTM), Università degli Studi di Milano, 20133 Milan, Italy; 4 Center for Studies on Bioispired Agro-Environmental Technology (BAT Center), Università degli Studi di Milano, 20133 Milan, Italy; 5 Department for the Innovation in Biological, Agrofood and Forestal Systems, Tuscia University, 01100 Viterbo, Italy; eovidi@unitus.it (E.O.); laghezzamasci@unitus.it (V.L.M.); antoniot@unitus.it (A.T.); 6 Department of Drug Chemistry and Technology, Sapienza University, 00185 Rome, Italy

**Keywords:** *Citrus medica*, fingered citron, separation, chemical analysis, volatile compounds, antibacterial activity, antiradical activity, cytotoxic activity, total flavonoid, total polyphenols

## Abstract

The present work aimed to chemically characterize and evaluate the antiradical power and biological effects of *Citrus medica* var. *sarcodactylus* essential oil (EO) and hydrolate (Hy) from exocarp as well as methanol extracts, from both exocarp and mesocarp (EEX and MEX). The whole fresh fruit was also investigated by SPME-GC/MS to describe its volatile composition. EO and Hy were analyzed by GC/MS and HS-GC/MS techniques, respectively. Limonene and *γ*-terpinene were found to be the most abundant compounds both in the fresh parts of the fruit and in the EO, while α-terpineol and terpinen-4-ol were in the Hy. The extracts were also rich in furan and coumarin derivatives. A good antiradical activity of all samples except Hy was detected both against ABTS·+ than DPPH·, removed up to about 50%. The antibacterial activity against *Bacillus cereus* and *Escherichia coli* was evaluated by microwell dilution method to determine MIC and MBC values. EEX and MEX showed efficacy at very high concentrations against both tested bacteria. The MIC value of EO against *B. cereus* was 0.5% *v*/*v*, while Hy was not able to inhibit the bacterial growth at the tested concentrations. Cytotoxicity investigated on the HL60 leukemia cell line by MTT assay provided an EC_50_ of 1.24% *v*/*v* for EO. Interesting activity of Hy was also observed.

## 1. Introduction

The *Citrus* species (Rutaceae) is the largest-spread fruit crop in the world, grown in more than 140 countries, of which China, Brazil, and the USA are the top producers [1,2]. They are widely used in the food sector, consumed as a fresh product for their strong fragrance, and in pharmaceutical, medicinal, and cosmetic fields thanks to the beneficial properties of their biologically active constituents [3,4]. In fact, multiple effects such as antioxidant, anti-inflammatory, anticoagulant, and anticancer qualities have been documented. Even essential oils (EOs), usually obtained from the citrus peel, being rich in secondary metabolites [5], are widely used in the food industry for conservation purposes or as additives to enhance the aroma [6], as well as in aromatherapy [7].

Buddha’s hand is the common name of *C. medica* var. *sarcodactylus* (Siebold ex Hoola van Nooten) Swingle (Rutaceae family). Native to northwestern India, it is a small tree with long and irregular branches full of thorns, known to produce peculiarly shaped fruits (up to 30 cm long), segmented into finger-like protrusions, characterized by a white, non-bitter, pulp-free mesocarp (albedo). Externally, the yellowish-orange exocarp (flavedo) is highly fragrant when ripe [8]. Both parts are edible, and are used to prepare desserts, drinks, candies, and sauces [9]. Recently, Buddha’s hand fruits have been frequently used in many Japanese restaurants in combination with peaches to serve a fruit salad, and more occasionally as ingredients to formulate crisps and cookies. In general, in the eastern countries where they are known as fingered citrons or as Fo Shou, the fruit is traditionally used in folk medicine. For example, in China, Buddha’s hand is appreciated as a tonic, antispasmodic, antiemetic, and expectorant, while in Taiwan it is used for the treatment of stomachache, headache, edema, rheumatism, infectious hepatitis, and arthritis. It is also taken as an adjuvant in the treatment of respiratory tract infections, asthma, and hypertension [10,11,12]. Some biological activities, including anti-inflammatory, antimicrobial, antioxidant, cytotoxic, and hypoglycemic effects of Buddha’s hand extracts or EOs obtained from different parts of the plant (fruits, leaves, peels, roots, stems) have been reported [10,13,14,15,16,17]. Likewise, its chemical composition has been indicated as showing the presence of classes of compounds such as coumarins, flavonoids, phenolic acids, terpenoids, and steroids [10,18].

In our study, we chemically investigated exocarp EO and hydrolate (Hy), as well as exocarp and mesocarp methanol extracts (EEX and MEX), never characterized up to now, with the aim to deepening the knowledge of the secondary metabolites (volatiles and not volatiles) of Buddha’s hand, as well as its bioactive potential.

## 2. Materials and Methods

### 2.1. Materials

Ethanol, methanol, 6-hydroxy-2,5,7,8-tetramethylchroman-2-carboxylic acid (Trolox), 2,2-Diphenyl-1-picrylhydrazyl (DPPH), 2,2′-azinobis (3-ethylbenzothiazoline-6-sulfonic acid) diammonium salt (ABTS), potassium persulfate (K_2_S_2_O_8_), Lysogeny Broth with Agar and Thiazolyl Blue Tetrazolium Bromide (MTT) were from Merck (Darmstadt, Germany). Gentamicin sulfate was purchased from Biochrome.

#### Plant Material

Buddha’s hand (*Citrus medica* var. *sarcodactylus*) was grown in the Nyon region (Switzerland) and supplied by the Niels Rodin’s Citrus Farm. Ripe fruit was harvested in October 2020 and stored in a fridge (about 5 °C) until analysis. Before use, it was washed with deionized water and the mesocarp was manually separated from the exocarp.

### 2.2. Hydrodistillation

EO and hydrolate of the Buddha’s hand were obtained by subjecting the exocarp (16 g) to hydrodistillation for 3 h using a clevenger-type apparatus. EO was dried over anhydrous sodium sulfate and kept in the fridge (4 °C) until use.

### 2.3. Extract Peparation

Exocarp and mesocarp of the Buddha’s hand fruit were extracted in agreement with Vitalini et al. [19]. Both tissues were pulverized in liquid nitrogen with a chilled mortar and pestle, then weighed and placed in MeOH (3 mL/g). The mixtures were sonicated and stirred for 10 and 20 min, respectively. Subsequently, each sample was decanted, and the supernatant centrifuged at 10,000 g for 10 min. The resulting extract was filtered through a 0.22 µm filter and combined with that of a second extraction. The solvent was removed in a rotary evaporator (RV 08-VC, IKA, Staufen, Germany) and the residues (EEX and MEX) were stored in glass vials at 4 °C until use. 

### 2.4. SPME Sampling of Exocarp and Mesocarp

To investigate the volatile chemical composition of the Buddha’s hand fruit without modification, thin slices of exocarp (~2 g) and mesocarp (~2 g) were individually placed into a 20 mL glass vial with PTFE-coated silicone septum. The sampling was performed by SPME technique following Vitalini et al. [20], with some modifications. An SPME device from Supelco (Bellefonte, PA, USA) with 1 cm fiber coated with 50/30 μm DVB/CAR/PDMS (divinylbenzene/carboxen/polydimethylsiloxane) was used. After an initial conditioning phase of the fiber, at 270 °C for 20 min, it was exposed to the equilibrated sample headspace for 30 min at 40 °C to capture the volatile compounds. After sampling, the SPME fiber was inserted into the GC injector maintained at 250 °C for the thermal desorption of the adsorbed compounds.

### 2.5. HS Sampling of Hy

To describe the chemical composition of the exocarp Hy vapor phase, a Perkin Elmer Headspace Turbomatrix 40 (Waltham, MA, USA) autosampler connected to GC-MS was used. The operative conditions have been previously described [21]. Briefly, about 2 mL of Hy was placed in a 20 mL vial sealed with headspace PTFE-coated silicone rubber septa and cap. The captured components from headspace of Hy were sent to the GC column via a transfer line maintained at a high temperature. 

### 2.6. GC-MS Analysis of EO and Hy

To characterize the chemical composition of EO and Hy from exocarp, a Clarus 500 model Perkin Elmer (Waltham, MA, USA) gas chromatograph coupled with a mass spectrometer and equipped with an FID (flame detector ionization) was used. Chromatographic separation was performed using a Varian Factor Four VF-1 capillary column and the gas carrier was He at flow rate of 1.0 mL min^−1^ in constant flow mode. The analytical conditions were applied following [22], with some modifications. For MS detection, an electron impact ionization (EI) system was used at 70 eV in scan mode in the range 35–400 *m*/*z*. The volatile separated compounds were identified by matching their mass spectra with those stored in the Wiley 2.2 and Nist 02 mass spectra libraries database and by comparison of their linear retention indices (LRIs), relative to C_8_–C_30_ n-alkanes analyzed under the same conditions, with those available in the literature. Relative concentrations of individual compounds were expressed as a percentage of the relative peak area to that of the total peak area without the use of an internal standard and any factor correction. All analyses were carried out in triplicate.

### 2.7. GC-MS Analysis of EEX and MEX

Chemical investigation of EEX and MEX was also performed through the same apparatus of Clarus 500. The dried extract and each sample were dissolved in 1 mL of methanol and the injection volume was 2 μL. The operative followed conditions have been reported previously [23]. 

### 2.8. Determination of Total Polyphenols

The total polyphenols of EEX and MEX were determined by the Folin–Ciocalteu method [24]. Briefly, a suitable aliquot of each extract was combined with 50 μL of Folin–Ciocalteau reagent. After 3 min, 100 μL of a saturated sodium carbonate solution was added and the final volume was made up to 2.5 mL with distilled water. The solutions were incubated in the dark for 1 h at room temperature, then their absorbance was read at 725 nm using a Jenway 6310 spectrophotometer (Keison, Chelmsford, Essex, UK). Tests were performed in triplicate and repeated three times. A calibration curve was prepared with gallic acid as standard at various concentrations (5–100 μg/mL) and the results were expressed as mg gallic acid equivalents (GAE) per g of fruit part. 

### 2.9. Determination of Total Flavonoids

The total flavonoid content of EEX and MEX was determined by the aluminum chloride colorimetric method [25], with some modifications. Briefly, 100 μL of each suitably diluted sample was separately mixed with 300 μL of methanol, 20 μL of 10% aluminum chloride, 20 μL of 1 M potassium acetate, and 560 μL of distilled water. The final solution was incubated in the dark for 30 min at room temperature. Then, the absorbance was measured at 420 nm using a Jenway 6310 spectrophotometer (Keison, Chelmsford, Essex, UK). A calibration curve was prepared with quercetin as standard at various concentrations (12.5–100 μg/mL). The test was performed in triplicate three times, and the results reported as mg of quercetin equivalents (QE) per g of fruit part.

### 2.10. DPPH Test

The radical-scavenging capacity of all samples (EO, Hy, EEX and MEX) against DPPH⋅ was assessed, as previously reported by Iriti et al. [26], with some modifications. Briefly, the DPPH⋅ solution was diluted with methanol to obtain 1.00 ± 0.03 absorbance units at 517 nm. Then, 10 μL of each EO were added to 1990 μL of this solution and vortexed. After a reaction time of 30 min in the dark at room temperature, the decrease in absorbance was read using a Jenway 6310 spectrophotometer (Keison, Chelmsford, Essex, UK) and expressed as RSA (radical scavenging activity)% = [(ABS_control_ − ABS_sample_)/ABS_control_] × 100. A DPPH⋅ solution without a sample was used as control. Tests were performed in triplicate.

### 2.11. ABTS Test

The ABTS⋅+ radical cation-scavenging activity was determined following Vitalini et al. [24]. The ABTS⋅+ radical cation was produced by reacting ABTS 7 mM with potassium persulfate 2.45 mM and keeping the mixture in the dark at room temperature for at least 6 h before use. Then, the ABTS⋅+ solution was diluted with ethanol to an absorbance of 0.7 (±0.02) at 734 nm and equilibrated at 30 °C. Then, 1 mL of this solution was mixed for 30 s with 10 μL of each sample (EO, Hy, EEX and MEX). Ethanol and a standard solution of the synthetic antioxidant 6-hydroxy-2,5,7,8-tetramethychroman-2-carboxylic acid (Trolox) were used as negative and positive controls, respectively. Their absorbance was read at 734 nm, at room temperature, 20 s after the end of the mixing using a Jenway 6310 spectrophotometer (Keison, Chelmsford, Essex, UK). Tests were performed in triplicate. The inhibition percentage was calculated, and the results expressed as radical scavenging activity (RSA) determined using the following equation: RSA (radical scavenging activity)% = [(ABS_control_ − ABS_sample_)/ABS_control_] × 100.

### 2.12. Antibacterial Activity

The antibacterial activity of EEX, MEX, EO, and Hy were defined by the Minimal Inhibitory Concentration (MIC) and the Minimal Bactericidal Concentration (MBC).

Gram-negative (*Escherichia coli* ATCC 25922) and Gram-positive (*Bacillus cereus* ATCC 10876) bacteria were obtained by growing cultures from the collection of the Plant Cytology and Biotechnology Laboratory (Tuscia University) and grown at 37 °C and 26 °C, respectively, in Lysogeny Broth (LB) agar. 

The microwell dilution method was used to determine Minimum Inhibitory Concentration (MIC), as reported by Garzoli et al. in 2021 [27]. All the matrices were diluted twelve times in LB broth (from 10 to 4.9 × 10^−3^ mg/mL and 1% to 5 × 10^−4^% for extracts and EOs, respectively), DMSO controls, growth controls without treatments, and sterility controls without bacteria were added to 96-microwell plates. As positive controls, gentamicin dilutions (100 µg/mL to 0.049 µg/mL) were used. In each well, a bacteria suspension (10^6^ CFU/mL) was added, except to the sterility control, and after 24 h, 20 µL of 3-(4,5-dimethylthiazol-2-yl)-2,5-diphenyltetrazolium bromide (200 µg/mL, MTT) was added to determine the bacterial growth.

To determine the Minimum Bactericidal Concentration (MBC), defined as the lowest concentration of an antibacterial agent required to kill a bacterium over a fixed period, 10 µL of the last four dilutions from the microwell dilution method in which no bacteria growth was observed were plated on LB agar following 24 h of incubation. The assay was carried out in triplicate. To define if the antibacterial activities of the tested samples were bactericidal or bacteriostatic, the ratio MBC/MIC of each sample was calculated and when the obtained value was <4, a bactericidal activity was defined. 

### 2.13. Cell Culturing and Cytotoxicity Test (MTT)

The cellular metabolic activity, as an indicator of cell viability of HL60 cells treated with extracts, EO, and related Hy of *C. medica*, was investigated by thiazolyl blue tetrazolium bromide assay (MTT assay), both in a dose- and time-dependent manner. The cells were maintained in a 75 cm^2^ flask containing RPMI1640 culture medium supplemented with 10% of FBS (fetal bovine serum), 1% glutamine and 1% penicillin/streptomycin, and maintained in an incubator to control temperature and %CO_2_ (37 °C and 5% CO_2_). Cells were seeded (2 × 10^4^ cells/well) in a 96-well plate 24 h before being treated. EO was dissolved in DMSO (50% *v*/*v*). Twelve two-fold diluted concentrations were applied from 0.5 to 0.01 mg/mL for extracts, from 1 × 10^−1^% to 2 × 10^−4^% *v*/*v* for the EO and from 50% to 1 × 10^−1^% *v*/*v* for the HY; the DMSO (0.05% final concentration) and ddH_2_O were used as solvent controls. As positive control, vinblastine sulfate (Merck KGaA, Darmstadt, Germany) was used. After treatments for 72 h, the medium has been removed and MTT solution (0.5 mg/mL) was added. Incubation was carried out for 3 h in a dark condition at 37 °C and, after formazan crystals solubilization by DMSO, the absorbance was read by a Tecan Sunrise™ UV-vis spectrophotometer at 595 nm. The obtained values were used to calculate the cell viability percentage (AAT Bioquest EC50 Calculator) [28], and to obtain the concentration at which the treatments exerted half of the maximal response values (EC50). Means ± SD were calculated by repeating the assay three times.

### 2.14. Statistical Analysis

All data were expressed as means ± standard deviation (SD). Statistical analyses were preformed using a one-way ANOVA test with a Stat-Plus software (AnalystSoft VC 2009), with the threshold of significance set at *p* < 0.05.

## 3. Results

### 3.1. Vapor Phase Chemical Composition

SPME-GC/MS analysis was performed to describe the volatile composition of fresh Buddha’s hand exocarp and mesocarp. In total, eighteen compounds were identified and listed in Table 1. In general, the monoterpene content largely prevailed over the sesquiterpene content in both the exocarp and mesocarp, and limonene (75.8%; 76.2%) and *γ*-terpinene (16.5%; 15.0%) were the most abundant compounds. *β*-Pinene (2.5%; 2.4%), *α*-pinene (1.6%; 1.4%), and cis-*β*-ocimene (1.2; 1.1%) were also present with lower and comparable relative percentages. Small qualitative differences were found between the two matrices; *β*-thujene (0.2%) and *α*-farnesene (0.2%) were present only in the exocarp while carveol (0.1%) and *α*-citral (0.1%) were present only in the mesocarp.

### 3.2. EEX and MEX Chemical Composition

The GC-MS analysis of the extracts allowed us to identify twelve compounds whose relative percentages are summarized in Table 2. Derivatives of furan were the main class of detected compounds, among which, 5-HMF was the main exponent with relative percentages of 14.7% and 24.8% in the EEX and in the MEX, respectively. 2-Furanmethanol (3.9%; 6.7%) and furaneol (3.1%; 3.6%) were also present in both extracts. Coumarin, 5,7-dimethoxy (50.6%) was the most abundant compound in the EEX followed by 2-pyrone (23.4%). On the contrary, 2-pyrone (33.1%) and 2,3-butanediol (23.7%) were the main component non-furanoic derivatives, present in MEX.

### 3.3. EO and Hy Chemical Composition

The content of volatile compounds for EO analysis was determined by GC-MS technique and by HS-GC/MS technique for Hy headspace analysis. In total, twenty-nine compounds were detected and listed in Table 3. The chemical composition of EO was found to be rich in monoterpene hydrocarbons (91.3%) followed by oxygenated monoterpenes (4.5%) and sesquiterpene hydrocarbons (3.9%). Conversely, Hy was rich in oxygenated monoterpenes (89.6%), while sesquiterpene hydrocarbons were absent. 

Qualitative and quantitative differences between the chemical composition of EO and HY were found. In detail, the volatile profile of EO was characterized by limonene (66.9%) and *γ*-terpinene (20.0%) as the most abundant compounds, while *α*-terpineol (44.7%) and terpinen-4-ol (21.6%) were in the Hy. 

Furthermore, several minor monoterpene components, such as *α*-thujene (0.2%), *α*-pinene (0.6%), *β*-thujene (0.1%), *β*-myrcene (0.9%), *β*-pinene (0.8%), (+)-4-carene (0.2%), R-(+)-citronellal (0.1%), nerol acetate (0.2%), and geranyl acetate (0.1%) found in the EO were missing in the Hy. Instead, other compounds such as *β*-terpinene (1.0%), linalol (5.7%), thymol (1.9%), and piperitenone (0.4%) were detected only in the HY.

### 3.4. Content of Polyphenols and Flavonoids

Table 4 shows the total polyphenol and flavonoid content of the methanolic extracts obtained from the Buddha’s hand exocarp and mesocarp. Both parts possessed a similar content of the two types of compounds. Nevertheless, as expected, EEX was found to be richer in both polyphenols and flavonoids than MEX. Their quantity in the EEX was about 1.5 times higher.

### 3.5. Antiradical Activity

Table 5 reports the activity values of all samples (EEX, MEX, EO and Hy) against the radicals ABTS·+ and DPPH·. As for their ability to scavenge ABTS·+, EEX, MEX and EO showed a similar efficacy, removing it by more than 50%. Hy was essentially inactive. The same trend was observed towards DPPH· by all samples except EO: it was able to inhibit this stable radical by only 26.4%.

### 3.6. EO, Hy, EEX and MEX Antibacterial Activity

The in vitro antibacterial activities of *C. medica* EEX, MEX, EO, Hy were tested using *B. cereus* and *E. coli* by defining MIC and MBC values. In Table 6, the obtained results for the extracts treatments are summarized. For *B. cereus*, EEX, MIC, and MBC values (2.5 and 5 mg/mL, respectively) were lower than MEX, MIC, and MBC values (10 mg/mL). For *E. coli*, the activity of the tested matrices was lower than the results for *B. cereus* (MIC values were 10 mg/mL for both extracts; MBC values were not attained at the tested concentrations).

In Table 7, the MIC value of EO was 0.5% and MBC value was 1.0% against *B. cereus*. For *E. coli*, MIC and MBC values were not attained. On the contrary, Hy did not possess activity at the tested concentration against both bacteria. MIC/MBC ratios defined that the active samples possessed bactericidal properties. 

### 3.7. Cytotoxicity Test on HL60 Cell Line

The cytotoxic activity of Buddha’s hand was evaluated for the EEX and MEX samples expressing the obtained EC_50_ values in mg/mL, and for EO and Hy samples expressing the obtained EC_50_ values in % *v*/*v* by using the MTT assay on the HL60 cell line. Treatments were carried out for 72 h and results are reported in Table 8 and Table 9. The EC_50_ value was 1.76 mg/mL for EEX, while the EC_50_ was not attained for MEX by the used concentration range. EO and Hy showed an EC_50_ of 1.24 and 2.97% *v*/*v*, respectively. 

## 4. Discussion

In this report, the volatile chemical composition of the fresh parts (exocarp and mesocarp) and of the respective methanol extracts, as well as of the hydrodistillation products (EO and Hy) obtained from the exocarp of *C. medica* var. *sarcodactylus*, grown in Switzerland, were described. The volatile profile of unmodified Buddha’s hand fruit, obtained by SPME-GC/MS analysis, highlighted an absolute prevalence of terpene hydrocarbons both in the exocarp and in the mesocarp with limonene and *γ*-terpinene as major compounds, according to Song et al. [29], who also considered this sampling technique as the most efficient to capture volatiles such as terpenes from this fresh matrix. Furthermore, it was also used to reveal the variation of the chemical composition of Buddha’s hand species due to the pickling processes [30]. 

There are a limited number of works on the chemical composition of EOs from Buddha’s hand fruit grown in other countries. In our study, exocarp EO was found to be rich in limonene and *γ*-terpinene, according to some previous studies [14,15,31,32,33,34]. Otherwise, other papers reported a lower content of these components [16] or, instead of *γ*-terpinene as the second major constituent, the presence of other molecules such as geraniol [11], *α*-pinene [35] and *β*-caryophyllene [36]. In general, such changes in the composition of EOs could be attributed to various factors, including the extraction or isolation method, conditions of analysis, geographic origin, climatic region conditions, and genetic variability [37,38,39,40,41]. With regard to Buddha’s hand EO, the stage of maturity is also a predominant factor. Wu et al. [16] reported that, in the mature phases, the quantity of monoterpenic hydrocarbons is higher, while the quantity of sesquiterpene hydrocarbons and oxygenated compounds is reduced.

To the best of our knowledge, this is the first study dealing with the chemical characterization of the EEX and MEX and of the Hy by GC/MS and HS-GC/MS techniques, respectively. The findings showed that extracts were rich in furan and coumarin compounds, while in the Hy, oxygenated monoterpenes dominated. 

In addition to these volatiles, the presence of polyphenols including flavonoids in both EEX and MEX could explain the biological activities recorded in this study. Polyphenols are widely recognized to be strong antioxidants, with the ability to neutralize free radicals [42]. Many phenolic compounds may also exhibit significant antibacterial activity involving some sites of action at the cellular level [43]. Previously, the antioxidant activity of Buddha’s hand EO has been investigated by obtaining variable values in relation to various considered factors, such as isolation methods, heat stress, or maturity stage, as well as the involved target [16,37,44]. Our data were in line with those of Wu et al. [16], who, among the test samples, reported the lowest scavenging ability against DPPH· for mature Buddha’s hand fruit. Nevertheless, the results of other works showed different DPPH radical-scavenging activities, with both lower and higher inhibition percentages [45,46]. The same goes for the activity detected towards ABTS·+ [45,47]. The discrepancy found could be explained by many factors, including those listed above, but also by different chemical compositions of the same *Citrus* species due to varied growing areas and climatic conditions. A large variety of EOs also possess the capacity to inhibit microbiological growth, and their potential applications are always the subject of research [48,49,50]. In our study, the antibacterial activity of the EEX, MEX, EO, and Hy of Buddha’s hand were investigated by the determination of MIC and MBC values against *B. cereus* and *E. coli*. All the samples were more active against *B. cereus* than *E. coli*; for the latter, MIC values were not attained at the tested concentrations except for the samples EEX and MEX, for which they were determined, although at a high concentration. Against *B. cereus*, EEX was more active than MEX. In particular, the highest activity recorded in EEX could be due to its major compound, coumarin, 5,7-dimethoxy, according to what was reported by Sahoo et al. [51]. EO had low a MIC value, showing high antibacterial activity. Previous studies have reported the antibacterial activity of *Citrus medica* L. var. *sarcodactylus* EO against food-borne bacteria, and various human multi-drug-resistant pathogenic bacteria [17,31,52]. Lou et al. [46] reported that the nano-emulsified EO showed an increased activity on *S. aureus* and on *E. coli* inhibiting the bacteria biofilm formation in tofu. Furthermore, the main constituents of EO, such as limonene and γ-terpinene, have been shown to have antimicrobial activity [53,54,55,56].

EEX was also found to have a cytotoxic effect against HL60 human cell line. This power could be attributed to its chemical composition. In fact, coumarins and coumarin derivatives possess a broad range of biological activities, including anticancer activity by apoptosis induction, cell cycle arrest, and several other mechanisms of action [57,58]. The 5-HMF compound has also been reported to have cytotoxic activity against HCT-8, A549, and SGC-7901 cell lines [59], as well as exert antioxidant activity [60]. In our investigation of the extracts, we also detected the 2-pyrone compound with considerable percentage values; previous studies have demonstrated both the antimicrobial and cytotoxic power of 2-pyrone derivatives [61].

In this work, the chosen techniques to investigate the volatile chemical composition and to study the biological activities, represent the first screening to be used [62,63,64,65]. The results obtained by EO and Hy MTT assay expressed an interesting cytotoxic activity. Buddha’s hand EO and limonene, its most abundant component, were shown to possess cancer-specific activity when tested against the skin melanoma A375 cells and normal skin cells [66]. Concerning Hy cytotoxicity, EC_50_ results were quite low, which revealed a very good activity. In our investigation, Hy was rich in *α*-terpineol and terpinen-4-ol, whose potential anticancer action, through cycle inhibition and apoptosis induction, has been demonstrated [67,68]. These compounds could explain the Hy cytotoxicity that has, until now, never been investigated.

## 5. Conclusions

In this multidisciplinary study, the chemical volatile composition of Buddha’s hand fresh parts, exocarp EO and Hy, and methanol extracts from exocarp and mesocarp were characterized by HS-SPME-GC/MS techniques. Furthermore, the content of polyphenols and flavonoids as well as antioxidant power and antibacterial and cytotoxic action were evaluated. The findings were particularly interesting regarding the cytotoxic effect against the HL60 human cell line shown both by the EEX and MEX, and by Hy, investigated for the first time. The obtained data could be useful as additional information on the phytochemical and biological properties of this fruit and, considering the large pool of bioactive detected compounds, highlight its functional benefits. Furthermore, the secondary identified metabolites could represent an environmentally friendly platform to produce new nutraceuticals usable in food, or healthy diets.

## Figures and Tables

**Table 1:** Chemical composition (percentage mean value ± standard deviation) of exocarp and mesocarp.

No.	Component ^1^	LRI ^2^	LRI ^3^	Exocarp ^4^ (%)	Mesocarp ^5^ (%)
1	*α*-thujene	923	925	0.5 ± 0.02	0.4 ± 0.03
2	*α*-pinene	941	943	1.6 ± 0.02	1.4 ± 0.01
3	*β*-thujene	966	968	0.2 ± 0.01	-
4	*β*-pinene	980	986	2.5 ± 0.02	2.4 ± 0.02
5	*α*-phellandrene	998	996	-	tr
6	*p*-cymene	1020	1016	0.2 ± 0.02	0.7 ± 0.03
7	limonene	1024	1023	75.8 ± 0.02	76.2 ± 0.02
8	cis-*β*-ocimene	1035	1032	1.2 ± 0.02	1.1 ± 0.02
9	*γ*-terpinene	1057	1054	16.5 ± 0.02	15.0 ± 0.02
10	*α*-terpinolene	1081	1078	0.2 ± 0.02	0.6 ± 0.02
11	*α*-terpineol	1185	1183	0.1 ± 0.01	0.4 ± 0.02
12	carveol	1205	1201	-	0.1 ± 0.00
13	4-terpinenyl acetate	1282	1286	0.5 ± 0.02	0.1 ± 0.02
14	*α*-citral	1285	1287	-	0.1 ± 0.00
15	*α*-bergamotene	1433	1431	0.3 ± 0.02	0.6 ± 0.02
16	*α*-himachalene	1451	1447	0.1 ± 0.02	0.6 ± 0.02
17	*γ*-gurjunene	1482	1479	-	tr
18	*α*-farnesene	1510	1506	0.2 ± 0.02	-
	SUM			99.9	99.7
	Monoterpene hydrocarbons			98.7	97.8
	Oxygenated monoterpenes			0.6	0.7
	Sesquiterpene hydrocarbons			0.6	1.2

^1^ The components are reported according to their elution order on apolar column; ^2^, Linear Retention Indices measured on apolar column; ^3^, Linear Retention Indices from literature; Exocarp ^4^, percentage mean values of exocarp components (%); Mesocarp ^5^, percentage mean values of mesocarp components; -, not detected; tr, traces (mean value < 0.1%).

**Table 2:** Chemical composition (percentage mean value ± standard deviation) of exocarp and mesocarp extracts.

No.	Component ^1^	LRI ^2^	LRI ^3^	EEX ^4^ (%)	MEX ^5^ (%)
1	2,3-butanediol	785	789	-	23.7 ± 0.02
2	furfural	796	794	1.8 ± 0.01	3.9 ± 0.03
3	2(3H)-furanone, 5-methyl-	832	830	0.9 ± 0.02	-
4	2-furanmethanol	860	856	3.9 ± 0.02	6.7 ± 0.04
5	furfural, 5-methyl-	970	965	-	1.9 ± 0.03
6	furaneol	1052	1055	3.1 ± 0.02	3.6 ± 0.01
7	2-pyrone	1130	1134	23.4 ± 0.02	33.1 ± 0.01
8	5-HMF	1210	1208	14.7 ± 0.02	24.8 ± 0.02
9	(E)-*β*-farnesene	1461	*	1.0 ± 0.02	-
10	(Z,E)-*α*-farnesene	1480	1475	0.6 ± 0.02	-
11	coumarin, 5,7-dimethoxy	1920	1916	50.6 ± 0.02	0.7 ± 0.02
12	hexadecanoic acid	1960	1954	-	1.6 ± 0.02
	SUM			100.0	100.0

^1^ The components are reported according to their elution order on apolar column; ^2^, Linear Retention Indices measured on apolar column; ^3^, Linear Retention Indices from literature; *, LRI not available; EEX ^4^, percentage mean values of exocarp components extract (%); MEX ^5^, percentage mean values of components mesocarp; -, not detected; tr, traces (mean value < 0.1%).

**Table 3:** Chemical composition (percentage mean value ± standard deviation) of EO and Hy.

No.	Component ^1^	LRI ^2^	LRI ^3^	EO ^4^ (%)	Hy ^5^ (%)
1	*α*-thujene	923	925	0.2 ± 0.02	-
2	*α*-pinene	941	943	0.6 ± 0.03	-
3	*β*-thujene	966	968	0.1 ± 0.00	-
4	*β*-myrcene	983	980	0.9 ± 0.01	-
5	*β*-pinene	980	986	0.8 ± 0.01	-
6	*α*-phellandrene	998	996	tr	-
7	(+)-4-carene	1005	1001	0.2 ± 0.02	-
8	*p*-cymene	1020	1016	1.6 ± 0.02	0.4 ± 0.01
9	limonene	1024	1023	66.9 ± 0.02	3.2 ± 0.02
10	*β*-terpinene	1040	1036	-	1.0 ± 0.01
11	*γ*-terpinene	1057	1054	20.0 ± 0.03	-
12	linalol	1091	1088	-	5.7 ± 0.03
13	R-(+)-citronellal	1158	1152	0.1 ± 0.02	-
14	terpinen-4-ol	1186	1182	0.5 ± 0.02	21.6 ± 0.02
15	*α*-terpineol	1185	1193	1.2 ± 0.01	44.7 ± 0.01
16	*cis*-geraniol	1228	1231	0.2 ± 0.02	7.2 ± 0.01
17	*β*-citral	1238	1242	0.9 ± 0.05	5.8 ± 0.03
18	*α*-citral	1285	1287	1.3 ± 0.01	8.0 ± 0.02
19	thymol	1308	1310	-	1.9 ± 0.02
20	piperitenone	1316	1315	-	0.4 ± 0.01
21	nerol acetate	1362	1365	0.2 ± 0.02	-
22	geranyl acetate	1381	1380	0.1 ± 0.02	-
23	*β*-caryophyllene	1455	1457	1.7 ± 0.02	-
24	humulene	1470	1473	0.1 ± 0.00	-
25	germacrene D	1491	1489	0.6 ± 0.01	-
26	*β*-bisabolene	1505	1501	1.2 ± 0.01	-
27	bicyclogermacrene	1510	*	0.1 ± 0.00	-
28	*δ*-cadinene	1532	1530	0.1 ± 0.00	-
29	*α*-bisabolol	1670	1668	0.1 ± 0.02	-
	SUM			99.7	99.9
	Monoterpene hydrocarbons			91.3	10.3
	Oxygenated monoterpenes			4.5	89.6
	Sesquiterpene hydrocarbons			3.9	-

^1^ The components are reported according to their elution order on apolar column; ^2^, Linear Retention Indices measured on apolar column; ^3^, Linear Retention indices from literature; *, LRI not available; EO ^4^, percentage mean values of exocarp components (%); Hy ^5^, percentage mean values of exocarp components (%); -, not detected; tr, traces (mean value < 0.1%).

**Table 4:** Total polyphenols and flavonoids of EEX and MEX.

Samples	Total Polyphenols (mg GAE/g Fruit Part)	Total Flavonoids (mg QE/g Fruit Part)
EEX	2.52 ± 0.07	2.20 ± 0.26
MEX	1.74 ± 0.02	1.50 ± 0.06

EEX, exocarp extract; MEX, mesocarp extract.

**Table 5:** Antiradical power of EEX and MEX.

Samples	ABTS RSA (%)	DPPH RSA (%)
EEX	55.8 ± 5.4	55.7 ± 1.20
MEX	52.0 ± 0.4	46.7 ± 0.82
EO	54.1 ± 0.2	26.4 ± 0.74
Hy	3.1 ± 0.2	2.5 ± 0.3

EEX, exocarp extract; MEX, mesocarp extract; EO, exocarp essential oil.

**Table 6:** Antibacterial activity of EEX and MEX.

	EEX			MEX		
Strains	MIC ^1^	MBC ^2^	MBC/MIC Ratio	MIC ^1^	MBC ^2^	MBC/MIC Ratio
*B. cereus*	2.5	5.0	2.0	10.0	10.0	1.0
*E. coli*	10.0	na	-	10.0	na	-

^1^ Minimal Inhibitory Concentration and ^2^ Minimal Bactericidal Concentration expressed in mg/mL. EEX, exocarp extract; MEX, mesocarp extract.

**Table 7:** Antibacterial activity of EO and HY.

	EO			Hy		
Strains	MIC ^1^	MBC ^2^	MBC/MIC Ratio	MIC ^1^	MBC ^2^	MBC/MIC Ratio
*B. cereus*	0.5	1.0	2.0	na	-	-
*E. coli*	na	-	-	na	-	-

^1^ Minimal Inhibitory Concentration and ^2^ Minimal Bactericidal Concentration expressed in % *v*/*v*; na, not attained.

**Table 8:** Cytotoxic activity of EEX and MEX on HL60 cell line for 72 h.

Samples	EC_50_ ± SD
EEX	1.76 ± 0.32
MEX	na

Effective Concentration (EC_50_ value ± SD) are expressed in mg/mL. EEX, exocarp extract; MEX, mesocarp extract.

**Table 9:** Cytotoxic activity of EO and Hy on HL60 cell line for 72 h.

Samples	EC_50_ ± SD
EO	1.24 ± 0.42
Hy	2.97 ± 0.07

Effective Concentration (EC_50_ value ± SD) are expressed in % *v*/*v* for EO and Hy. EO, exocarp essential oil; Hy, exocarp hydrolate.

## Data Availability

All generated data are included in this article.

## Sample Availability

Samples of the Buddha’s hand are available from the authors.
